# Simplified propofol-based deep sedation by electrophysiologists: Safe and versatile for pulsed field and thermal ablation

**DOI:** 10.1016/j.hroo.2026.02.019

**Published:** 2026-02-28

**Authors:** René H. Worck, Signe M. Hansen, Lone Tøttrup, Tina Aagaard, Xu Chen, Uffe J. Gang, Jim Hansen, Morten L. Hansen, Peter K. Jacobsen, Arne Johannessen, Christian Jøns, Niels C. Sandgaard, Martin H. Ruwald

**Affiliations:** Copenhagen Heart Center, Rigshospitalet and Gentofte Hospital, Copenhagen, Denmark

**Keywords:** Deep sedation, Pulsed field ablation, Safety, Versatility, Resources, Laboratory occupancy time

## Abstract

**Background:**

Pulsed field ablation (PFA) induces substantial procedural pain and typically requires deep sedation or general anesthesia. However, limited anesthesia resources constrain broad PFA adoption.

**Objective:**

We evaluated a simplified propofol-based deep sedation (s-DS) protocol for its safety, feasibility, and adaptability across various ablation modalities.

**Methods:**

Between May 2023 and June 2025, 497 consecutive patients underwent ablation under an s-DS regimen (propofol infusion and low-dose fentanyl only) by the electrophysiology laboratory staff. Procedures included PFA (n = 403) and thermal ablation (n = 94) across diverse arrhythmia types. Sedation parameters, hemodynamics, and recovery metrics were prospectively recorded. The study was structured in 2 phases: implementation phase (phase 1 n = 144) and routine adoption (phase 2 n = 353). Time to the ward (TTW) served as a surrogate of team proficiency and workflow efficiency.

**Results:**

s-DS was feasible and safe. Urgent conversion to general anesthesia occurred in 1 patient (0.2%), and transient mask ventilation was required in 9 (1.8%). PFA required higher fentanyl doses (*P* < .001), induced greater mean arterial blood pressure reductions (*P* = .004), and longer TTWs (*P* < .001) than thermal ablation, whereas oxygen saturation and respiratory support were comparable. TTW significantly declined with increasing operator and nursing experience (*P* < .001). No major adverse events, airway complications, or late respiratory failures occurred.

**Conclusion:**

s-DS by electrophysiology laboratory staff enabled safe, versatile, effective, and pain-free ablations across all energy sources and arrhythmia types. s-DS offered a resource-efficient workflow with short TTW and reduced the need for prolonged postprocedure observation.

**Key Findings**

▪ Simplified propofol-based deep sedation (s-DS) can be administered effectively and safely by a trained team of nurses and electrophysiologists with access to anesthesiologic expertise on immediate call.
▪ s-DS provides versatile sedation that can be tailored for both thermal and pulsed field ablation.
▪ s-DS allows short postprocedure time to the ward, which may reduce laboratory occupancy time and reduce the requirement of prolonged postprocedure observation.

## Introduction

Effective sedation and pain management are critical for achieving durable lesions in cardiac ablation. Pulsed field ablation (PFA) is considerably more painful than thermal ablation with cryoballoon (CRYO) and radiofrequency (RF) and therefore requires deeper sedation. However, increasing adoption of PFA conflicts with limited access to general anesthesia (GA) and anesthesia support for deep sedation (DS).^1^ Contrary to GA, traditional DS combines propofol, benzodiazepines, and opiates while maintaining spontaneous ventilation without endotracheal intubation (eIT) and has been used safely by electrophysiologists for more than 2 decades in thermal ablations and more recently in PFA.^2^^,^^3^ Nevertheless, skepticism and organizational barriers continue to limit its wider use, primarily owing to concerns about maintaining oxygenation and handling acute airway interventions including urgent eIT during procedures.

Although effective, the triple-drug approach in DS may be unnecessarily complex and increase the risk of respiratory depression.^4^ Propofol, the cornerstone sedative, has ancillary qualities, exerts intrinsic analgesic properties, and acts synergistically with opioids, raising the question of whether benzodiazepines are required. Building on this rationale, we developed a simplified propofol-based DS (s-DS) protocol limited to minor doses of opioids and continuous propofol infusion, omitting the benzodiazepines.^5^ Recent reports from atrial fibrillation (AF) and ventricular tachycardia ablation suggest the feasibility of such simplified regimens, but adoption has been limited.^6^^,^^7^

To evaluate whether propofol-based s-DS was safe, effective, and adaptable for routine implementation, we prospectively registered all ablation procedures performed in s-DS. This report describes the implementation (phase 1) and subsequent routine adoption (phase 2) for ablations with pulsed field (PF) (n = 403) and thermal energies (n = 94) across diverse cardiac tachyarrhythmias.

## Methods

Between May 2023 and June 2025, in aggregate, 2054 patients were a priori considered eligible for GA. Of these, 497 patients were sedated using the s-DS protocol for cardiac ablation and were prospectively registered. Inclusion was mainly determined by organizational and logistical factors such as availability of GA or s-DS–suitable catheterization laboratories and availability of s-DS–trained personnel, but also by the share of patients fulfilling the inclusion criteria (Table 1) and their willingness to consent. The s-DS program was structured in 2 phases: an early implementation phase (phase 1 n = 144) and a subsequent routine adoption (phase 2 n = 353). In phase 1, detailed pharmacologic, hemodynamic, respiratory, and procedural data were collected, allowing comparison of s-DS performance between thermal ablation and PFA. Based on favorable phase 1 safety outcomes, the inclusion criteria for s-DS were slightly adjusted for phase 2. The time from the procedure end to patients being awake and cleared for basic ward observation (time to the ward [TTW]) was used as a surrogate of s-DS team proficiency in delivering effective yet balanced sedation and compared between phases.

**Table 1:** Inclusion criteria to undergo s-DS

BMI ≤33 kg/m^2^
No previous anesthesia or sedation complications
ASA class ≤2 (stable IHD, LVEF <45%, implanted device, and remote stroke accepted despite ASA class = 3)
No active substance abuse (opiates, sedatives, alcohol, and others)
Anticipated procedure time ≤3 h
No obstructive sleep apnea, COPD, or asthma
No known reflux to the esophagus
No active GLP-1 analog treatment that delays gastric emptying
LVEF ≥35%

ASA = American Society of Anesthesiologists; BMI = body mass index; COPD = chronic obstructive pulmonary disease; GLP-1 = glucagon-like peptide-1; IHD = ischemic heart disease; LVEF = left ventricular ejection fraction; s-DS = simplified propofol-based deep sedation.

### Study population

497 consecutive patients were referred for cardiac ablation and sedated in s-DS. Eligibility for s-DS required a thorough review of patients’ charts to match the inclusion criteria that focused on airway, pulmonary, and circulatory comorbidities (Table 1). Reasons for a lack of suitability for s-DS were not systematically registered in the database; however, the most frequent causes of exclusion were severe obesity with a body mass index of >33 kg/m^2^, known sleep apnea, gastric-esophageal reflux, and the use of glucagon-like peptide-1 analogs delaying gastric emptying since avoiding the risk of aspiration was paramount. Informed consent was obtained from all participants. Institutional review board approval was waived owing to the use of deidentified data. The research reported in this paper adhered to the Declaration of Helsinki as revised in 2024.

### Personnel

s-DS was delivered by at least 2 qualified professionals, including the operator electrophysiologist. At the outset of phase 1, sedation was handled exclusively by 2 electrophysiologists with nurses in observing and documenting roles. After a short learning period, practical sedation tasks were transferred to dedicated s-DS nurses, who managed the protocol autonomously in close dialogue with the operator (Supplement 1).

### Sedation protocol and monitoring

On arrival, the inclusion criteria for s-DS were confirmed, including the simplified airway risk index^8^ to anticipate potential need for noninvasive ventilation (NiV) or eIT. If the simplified airway risk index indicated potential problematic airway access, then scheduled s-DS was cancelled, and the patient was offered conscious sedation or GA. Sedation workflow and drug dosing are presented in Supplement 2 and 3. Briefly, all actions, measurements, and monitoring were documented in real time in anesthesia charts or the electronic patient journal. This included drug administration, continuous electrocardiogram, capnography, oxygen (O_2_) saturation, respiratory depth/frequency, and noninvasive blood pressure every 2–3 minutes. Sedation was initiated with a small fentanyl bolus immediately followed by propofol: bolus and start infusion. O_2_ (3–5 L/min) was supplied. Steady-state sedation was defined by correct airway placement without irritation, adequate spontaneous ventilation, O_2_ saturation of >93%, and mean arterial pressure of ≥60 mm Hg, maintaining a Richmond agitation-sedation scale (RASS) score of −4 to −5.^9^ Sedation depth and hemodynamics were continuously adjusted with propofol, fentanyl, intravenous volume supplement, and phenylephrine as needed. Most patients received ondansetron to prevent nausea. Propofol infusion was stopped ∼5 minutes before the anticipated procedure-end to allow rapid awakening. This protocol supported efficient laboratory turnover and potentially reduced the need for intensive postprocedure observation. Recovery was assessed by RASS scoring, and once RASS scores of −1 or 0 and TTW were documented, patients were transferred directly to the normal ward, accompanied by the s-DS nurse for handover.

### Ablation procedures

Most lesion sets ablated under s-DS were pulmonary vein isolation (PVI) or “PVI+,” denoting any instance of additional linear or punctuate ablation, posterior wall isolation, ectopic triggers, linear, or point-ablation to treat reentry or other atrial tachycardia. The remaining lesion sets included cavotricuspid isthmus block for cavotricuspid isthmus–dependent tachycardias, supraventricular tachycardias, ventricular arrhythmias (premature ventricular contractions and ventricular tachycardia), and “other” such as sinus node modification, His bundle ablation, and ablations not categorized elsewhere.

PVI and PVI+ procedures were performed using PFA, RF ablation (RFA), or CRYO ablation, applied in both single-shot (SS) and point-by-point (PP) configurations, except for CRYO ablation—done exclusively as SS.

Procedure workflows for PFA (SS and PP), RFA (SS and PP), and CRYO ablation at our center were published previously.^10^, ^11^, ^12^, ^13^ For supraventricular tachycardias ablated near the atrioventricular node, only RFA was used, whereas all other locations were ablated with RFA, PFA, or both. Specific ablation systems and catheters used are detailed in Supplement 4. In brief, PP-PFA was delivered using the Galvanize/CardioFocus platform, SS-PFA with the Farastar/Farawave platform, PP-RFA with the Smartablate, SS-RFA with the nGEN, and CRYO ablation with the CryoConsole generators exclusively.

### Statistical analyses

Categorial data are presented as means and percentages and compared using the χ^2^ test. Normally distributed continuous data are presented as means ± standard deviations and compared using the Student *t* test. Non-normally distributed continuous data are presented as median and interquartile range and compared using Wilcoxon’s signed rank test. *P* < .05 was considered statistically significant.

## Results

All 497 consecutive patients who underwent s-DS for cardiac ablation were included. Given that most patients received ablation for AF and AF-related arrhythmias, the patient cohort resembled typical AF cohorts regarding age and gender. Baseline characteristics and comorbidities of phase 1 patients are presented in Table 2. Only 25% were registered as hypertensive. During implementation (phase 2), patient characteristics were not as systematically collected as during phase 1. However, basic demographics such as distribution of age, gender, and body mass index were comparable between phases, as presented in Table 3. Moreover, the distribution of treated arrhythmias was similar between phases, as presented in Table 3. The course of s-DS differed significantly depending on whether thermal ablation or PFA was used, as presented in Table 4. Over time, the most prevalent ablation mode evolved from PP-PFA in phase 1 to SS-PFA in phase 2 (Table 3).

**Table 2:** Baseline characteristics of phase 1 patients

N = 144	
Male gender, %	69
Age, y, median (interquartile range)	66 (58–73)
BMI, kg/m^2^, mean ± standard deviation	24.9 ± 2.7
LVEF, n	
<40%	1
40%–59%	33
≥60%	110
Comorbidity, %	
Hypertension	25
IHD∗	3
CHF (NYHA class ≥II)	31
CHA_2_DS_2_-VAsc score,† n	
NA‡	44
1	40
2	30
3	17
≥4	13

BMI = body mass index; CHF = congestive heart failure; IHD = ischemic heart disease; LVEF = left ventricular ejection fraction; NA = not available; NYHA = New York Heart Association.

∗ Known and treated IHD.

† Heart Rhythm Society/European Heart Rhythm Association stroke-risk score in patients with atrial fibrillation or atrial flutter.

‡ 0 or not diagnosed as having atrial fibrillation or flutter.

**Table 3:** Lesions and ablation modes in phases 1 and 2 of s-DS

Variable	Implementation phase 1 (n = 144)	Routine phase 2 (n = 353)	*P* value
Male sex, %	69	68	1
Age, y	66 (58–73)	64 (55–71)	1
BMI, kg/m^2^	24.9 ± 2.7	25.4 ± 3.3	.12
Fentanyl, total, μg	102 ± 39	104 ± 31	.65
Propofol, total, mg	940 (714–1275)	880 (690–1178)	.23
TTW,∗ min	31 (24–41)	26 (21–34)	<.001
Lesion set†			
Ablation mode‡			

BMI = body mass index; CTIB = cavotricuspid isthmus block; PF = pulsed field; PP = point by point; PVI = pulmonary vein isolation; PVI+ = PVI + any instance of additional linear or punctuate ablation, posterior wall isolation, ectopic triggers, or line or point ablation to treat macroreentry or other atrial tachycardias; RF = radiofrequency; s-DS = simplified propofol-based deep sedation; SS = single shot; SVT = supraventricular tachycardia; TTW = time to the ward; VA = ventricular arrhythmia.

∗ Time from sheaths out/procedure end to patient leaving for basic observation on the ward.

† Other lesion set: linear or punctate ablations not related to PVI, that is, scar flutters, sinus node ablation, or His bundle ablation.

‡ Other ablation mode: cryoballoon, RF + PF, or not ablated.

**Table 4:** Thermal ablation vs PFA in s-DS phase 1

Variable	Ablation energy		*P* value
	Thermal (49)	PFA (93)	
Age, y	65 (59–73)	66 (33–85)	.89
Male gender, %	34 (69)	65 (70)	.95
BMI, kg/m^2^	24.4 ± 2.9	25.1 ± 2.6	.15
Start SBP, mm Hg	130 (122–142)	135 (125–150)	.12
Start DBP, mm Hg	75 (70–88)	76 (70–85)	.92
Start MAP, mm Hg	97 (81–103)	97 (90–103)	.43
Maximum Δ SBP, mm Hg	−45 (37–57)	−55 (40–71)	.013
Maximum Δ DBP, mm Hg	−25 (20–32)	−30 (23–40)	.019
Maximum Δ MAP, mm Hg	−32 (25–40)	−38 (30–47)	.004
Start O_2_ saturation, %	100 (99–100)	100 (99–100)	.71
Maximum Δ O_2_ saturation, %	−2 (0–4)	−3 (1–5)	.32
Maximum O_2_ flow, L/min	4 (3–5)	4 (3–5)	.16
Mandibular lift required, %	16%	5%	.03
NiV required, n/N	4%	1%	.57
Procedure time, min	82 (62–119)	79 (50–100)	.143
Propofol, total, mg	860 (719–1211)	962 (714–1314)	.126
Propofol rate, mg/kg × h	8.1 (6.9–9.9)	9.7 (8.4–11.5)	<.001
Fentanyl, total, μg	75 (50–100)	100 (100–125)	<.001
Fentanyl rate, μg/h	55 (37–73)	86 (67–111)	<.001
Phenylephrine, total, μg	0 (0–100)	175 (0–500)	.002
TTW,∗ min	25 (22–36)	35 (27–44)	<.001

Respiration, circulation, and drug use for s-DS in phase 1 (thermal vs PFA).

BMI = body mass index; DBP = diastolic blood pressure; MAP = mean arterial pressure; NiV = noninvasive ventilation; O_2_ = oxygen; PFA = pulsed field ablation; SBP = systolic blood pressure; SDP = diastolic blood pressure; s-DS = simplified propofol-based deep sedation; TTW = time to the ward; Δ = change of value vs before sedation.

∗ Time from the procedure end to the intensive observation end.

### Feasibility and safety

Safety and feasibility parameters for all s-DS procedures were encouraging, as presented in Table 5. (1) Of 497 initiated s-DSs, only 3 patients (0.6%) were converted to conscious sedation shortly after initiation owing to concern about breathing patterns, the ability to prolong the maintenance of satisfactory alveolar ventilation, and adequacy of per-protocol drug dosing. (2) In 9 patients (1.8%), transient—usually less than 5 minutes—hand-held mask ventilation (NiV) was deemed necessary by the personnel until sedation steady state was achieved and adequate spontaneous respiration was restored. No mechanical noninvasive respiratory support such as continuous or bilevel positive airway pressure was used for NiV. (3) 1 patient (0.2%) required urgent anesthesiologic intervention, eIT, and continuation of the procedure in GA. Of minor observations, 1 patient (0.2%) was transiently unresponsive and confused during recovery despite normal blood pressure and O_2_ saturation. He recovered spontaneously, was neurologically intact, and was discharged on the same day as planned. Three patients (0.6%) required significantly longer observation in the electrophysiology (EP) laboratory and prolonged TTW because blood pressure support with phenylephrine and volume/isotonic saline was required after the procedure. Finally, 1 patient (0.2%) was oliguric for 10 hours after the procedure. This case was associated with deeper and more prolonged intraprocedural hypotension than average, resolved spontaneously on oral hydration with full recovery and discharge 1 day after the procedure.

**Table 5:** Safety of s-DS

Parameter	Phase 1 (n = 144)	Phase 2 (353)	All (N = 497)
Required mandibular or other airway handling,∗ %	12 (8.3)	9 (2.5)	21 (4.2)
s-DS aborted, continued in conscious sedation	1 (0.6)	2 (0.5)	3 (0.6)
s-DS aborted, opportunistic conversion to GA, %	0	2 (0.6)	2 (0.4)
NiV,† %	3 (2)	6 (1.6)	9 (1.8)
Urgent eIT with conversion to GA, %	0	1 (0.3)	1 (0.2)
Hypotension after the procedure, %	1 (0.7)	2 (0.5)	3 (0.6)
Confusion after the procedure, %	0	1 (0.3)	1 (0.2)

GA = general anesthesia; eIT = endotracheal intubation; NiV = noninvasive ventilation; s-DS = simplified propofol-based deep sedation.

∗ Transient manual mandibular lift required to prevent or treat desaturation (arterial oxygen <93%) without need for mask ventilation.

† Noninvasive manual mask ventilation required.

Overall, s-DS was appreciated as safe by the involved professionals. However, balanced s-DS required continuous attention and prompt reaction to coughing, respiration, and blood pressure according to protocol (S2 and S3). Characteristic for phases 1 and 2 alike was that endocardial PFA near the left and right main bronchi, that is, near the roof of both the left and right pulmonary vein ostia, frequently induced bouts of coughing, which—with occasional diaphragmatic stimulation—rarely induced map shifts, for example, during PP-PFA based on CARTO anatomic mapping. SS-PFA with Farawave typically induced more phrenic capture and diaphragmatic movement than PP-PFA, but because these procedures were performed using fluoroscopy only, map shift was not a problem. With properly titrated drugs, we registered minimal body movement (other than respiratory) at a low level not discernible from patients ablated under GA without neuromuscular blockade. Repeat coughing was usually preventable with small boluses of propofol, but in the single patient who ultimately required urgent eIT, a prolonged bronchospasm was triggered by such PF-induced airway irritation. This procedure continued in GA without any sequelae. Thermal ablation did not induce cough under s-DS.

Detailed dosing of propofol, fentanyl, and phenylephrine to prevent or treat hypotension is listed for phase 1, along with the impact on blood pressures and arterial O_2_ saturation, and compared between thermal (less painful) and PFA energies (more painful) (Table 4). PFA required deeper levels of sedation than thermal ablation, as shown by significantly higher dosing of propofol and fentanyl. Accordingly, blood pressure reduction (delta values) and the ensuing need for vasoconstriction with phenylephrine were higher assessed both as the proportion of patients requiring phenylephrine (61% during PFA vs 37% during thermal ablation; *P* < .01) and as the cumulated required dose of phenylephrine (*P* = .002) (Table 4).

Spontaneous respiration is innately affected by propofol + opioid administration and thus often required minor manipulations with head and neck repositioning or oropharyngeal or nasal airway tube (or both) to achieve satisfactory spontaneous alveolar ventilation estimated qualitatively but reliably and operationally by continuous capnography using mask-mounted sensors. O_2_ saturation was only mildly affected by s-DS and was readily maintained within normal physiological range using moderate O_2_ supplementation and active titration of propofol infusion. In 16% of thermal compared with 5% of PFAs, transient mandibular manipulation was necessary to facilitate spontaneous ventilation and keep O_2_ saturation at more than 93% as required per protocol (S2). However, despite the deeper sedation required for PFA, measured O_2_ saturation and required O_2_ supplementation were similar between groups. TTW was significantly longer after PFA than thermal ablation (*P* < .001).

### Organizational learning

**Figure 1:**
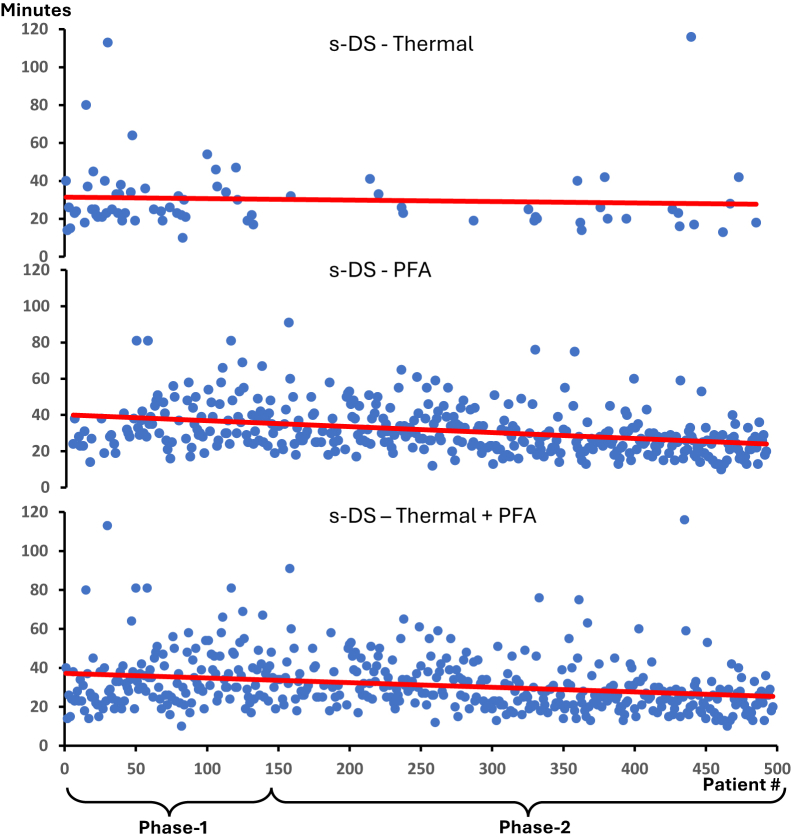
Time from the procedure end to transfer to the regular ward (TTW)—development over time and patient #. *Top:* TTW after thermal ablation. *Middle:* TTW after PFA. *Bottom:* TTW entire cohort. PFA = pulsed field ablation; s-DS = simplified propofol-based deep sedation.

TTW as a surrogate for s-DS optimization declined steadily over time, as well as the number of procedures (Figure 1), and was significantly shorter in phase 2 than phase 1 (*P* < .001) (Table 3). Despite early transfer from the EP laboratory to the general ward, no instances of “too early” takeover or any late relapse of circulatory or respiratory insufficiency with escalation of care were reported.

## Discussion

Implementation of PFA is limited by restricted access to GA and DS in many countries, sparking renewed interest in alternative sedation strategies. In this single-center experience, the implementation and routine adoption of s-DS conducted in 497 patients by electrophysiologists and trained catheterization laboratory nurses were safe and feasible across arrhythmias, lesion sets, and energy platforms. These included RFA, CRYO ablation, and PFA delivered in “single- shot” and “point-by-point” modes using the only 2 PFA platforms broadly available at the time. Thus, the favorable tolerability of PFA under s-DS reported here may not be extrapolated to later introduced PFA platforms. This cohort was a selected population. For instance, the prevalence of hypertension was lower than expected in a predominantly AF cohort, which may partly be caused by underreporting by patients, but selection bias, low comorbidity, and the fact that almost a fifth of the patients were treated for arrhythmias other than AF may also play a role. s-DS was associated with urgent conversion to GA in only 0.2% (1 patient) and seemed to carry logistical advantages by reducing the need for resource-demanding intensive postprocedure monitoring in specialized wards, which is otherwise routinely used for post-GA observation.

### Feasibility and safety

s-DS was feasible and safe across procedure types and ablation energies. As expected, PFA required higher sedative and analgesic dosages than thermal ablation to achieve sufficient pain-free sedation levels. This aligns with the findings by Galuszka et al^14^ who observed increased drug requirement and hemodynamic instability during PFA compared with thermal ablation. The higher dosages explain the more pronounced blood pressure reduction and the need for markedly higher doses of α_1_-stimulation with phenylephrine during PFA than thermal ablation. Drug doses were comparable with those of Rillig et al^6^ using an s-DS–like sedation protocol and with the large observational cohort of Salukhe et al^15^ reporting a mixed DS and s-DS–like regimen. However, they reported prolonged drug-induced hypotension in 14% compared with 0.6% in the current s-DS regimen. Apart from being a simpler model with only 2 analgesic-sedative drugs, the favorable safety of s-DS may partly be explained by our systematic use of α_1_-stimulation and active volume therapy with isotonic saline in the case of blood pressures of less than 60 mm Hg (mean arterial pressure cutoff).

The most feared adverse event during DS and s-DS is inducing a state of “irreversible” hypoventilation and failure to ventilate a patient with a collapsed or spasmic airway. Here, the availability of anesthesiologic expertise on short notice in an established collaboration with acute eIT is critical for patient safety. Foerschner et al^16^ reported that NiV was required in 1.5% of a large cohort undergoing thermal ablation for AF under triple-drug DS and only 0.03% requiring emergency eIT. In comparison, NiV was required in 1.8% under the current s-DS protocol, whereas urgent eIT was required in 1 patient (0.2%), and although the 2 cohorts are not directly comparable, it seems that both protocols were safe with insignificant minor differences despite deeper sedation being required in the current cohort given that most patients were subjected to (painful) PFA.

### s-DS organizational learning and logistics

TTW denotes the time from the procedure end to the patient leaving the laboratory for nonintensive monitoring at the regular ward with an RASS score of −1 or 0, stable hemodynamics, and respiratory function on their own physiological conditions. TTW might be a reasonable surrogate for aggregate team s-DS skills: among several factors TTW include (1) balancing of sedation, that is, sufficient but avoiding “overshoot” sedation; (2) communication in the team, for example, predicting the end of the procedure for early reduction of sedation; (3) uniform and precise scoring of patients’ awareness level using the RASS score; and (4) stable, conscious patients transferred to the regular ward with no late adverse effects from s-DS. The steady and significant decline of TTW over time may reflect a team-learning trajectory for s-DS. However, TTW is a crude metric with potential biases because it was not stratified by, for example, procedure complexity, ablation mode, or operator experience. Thus, whether aggregate logistics for s-DS carries true advantages vs GA may warrant formal scrutiny. Given that time consumption for vascular access, EP study, mapping, and ablation has evolved quite homogeneously and streamlined, optimizing TTW is critical to decrease total laboratory occupancy time—a metric that was regrettably not systematically recorded here. Recently, Rillig et al^6^ showed a significant 21% reduction in laboratory occupancy time for s-DS compared with GA for AF-related atrial PFA using the Sphere-9 catheter. Thus, s-DS performed by skilled staff might contribute to the optimization of EP laboratory logistics.

### Patient experience

The patient experiencing transient oliguria associated with procedural hypotension felt diffusively uncomfortable but recovered spontaneously. Otherwise, there was no negative feedback from patients after s-DS. However, patient-experience data were not systematically collected. Münkler et al^17^ reported high patient satisfaction after triple-drug DS for RFA, whereas Sciacca et al^18^ recently used a triple-drug DS protocol and found that 97% of patients were willing to undergo DS again in subsequent procedures. Positive patient experience and acceptance are mandatory for further adoption and utilization of balanced DS, hence optimizing the workflow of PFA.

### Regulatory considerations

Because of its potential to induce apnea and circulatory collapse with no specific reversal agent, propofol sedation by nonanesthesiologists is controversial albeit not directly prohibited across different jurisdictions, to the best of our knowledge. Therefore, it is pivotal that propofol-based sedation regimens are developed, implemented, and administered in close collaboration with a certified anesthesiologist who must be available on immediate call. Strict adherence to formal training programs for the staff performing the sedation to possess and demonstrate proper expertise is paramount to avoid harming patients and ensuing legal risk.

### Limitations

Several limitations of this study should be mentioned. First, this was a single-center, nonrandomized observational experience in selected patients of a high-volume center. Thus, findings may not be directly transferable to other centers. Second, although patients’ basic demographics were similar between phases, the lack of potentially important baseline parameters during implementation (phase 2), for example, left ventricular ejection fraction and prevalence of ischemic heart disease, may limit the generalizability of the findings. Third, despite positive feedback from patients and nurses providing postprocedure care, patient experiences and eventual delayed adverse reactions to s-DS were not systematically registered, prohibiting conclusions on these matters. Fourth, urgent intubation by an anesthetist was required in 0.2%—a small risk that can probably never be eliminated in s-DS without intubation. Thus, the presence of in-house anesthesia backup on immediate call remains mandatory, which may limit the adoption of s-DS. Moreover, the important issue of fluoroscopy use and the putative impact of s-DS vs GA was not addressed. The CHA_2_DS_2_-VAsc score for phase 1 patients with AF was maintained because the European Society of Cardiology (2024) revision to CHA_2_DS_2_-VA scoring had not yet been implemented.

## Conclusion

In this study, including 497 patients, s-DS was feasible, safe, and effective in providing pain-free ablations across energy sources and arrhythmia types provided (1) careful patient selection, (2) sedation by a dedicated and formally trained team of nurses and electrophysiologists, and (3) established collaboration and availability of anesthesiologic expertise on immediate call. Intensive observation during postprocedure recovery was completed in the procedure room, was short term, decreased over time, and reduced the need for observation in a recovery ward.

## Data availability statement

Anonymized data files of this study can be made available upon reasonable request to the corresponding author

## Disclosures

R.W. received speaker honoraria from Biosense Webster and Boston Scientific. X.C. received speaker honoraria from Abbott. J.H. received speaker and consulting honoraria from Boston Scientific, Biosense Webster, and CardioFocus. P.K.J. received speaker honoraria from Biosense Webster, Abbott, and Boston Scientific. M.R. received speaker honoraria from Biosense Webster and Boston Scientific. The other authors have no conflicts of interest to disclose.

## Footnotes

Appendix Supplementary data associated with this article can be found in the online version at https://doi.org/10.1016/j.hroo.2026.02.019.
