# Supplementary material for: Simplified propofol-based deep sedation by electrophysiologists: Safe and versatile for pulsed field and thermal ablation
Source: Heart Rhythm O2. 2026 Feb 28;7(5):872–9. doi: 10.1016/j.hroo.2026.02.019 (PMC13198331; doi:10.1016/j.hroo.2026.02.019)
Supplement: Supplementary File [file mmc1.docx]

**SUPPLEMENT S1: Simple Deep Sedation Personnel requirements and utilized ASA classification**

- s-DS for cardiac ablation requires constant presence of at least two sedation-qualified professionals.
- At least one of these professionals must be the physician who is ultimately responsible for the s-DS.
- The other professional is either another qualified physician or a qualified cardiology nurse who is specifically trained for s-DS
- One of the two professionals has the sole task of paying attention to the patient during s-DS.
- Another s-DS-qualified physician must be available to be summoned on short notice.
- The physician must demonstrate extensive insight into the pharmacokinetics and dynamics of the drugs involved, be experienced in the use of s-DS and have documented ability to handle respiratory problems and vascular complications.
- In complex procedures, the presence of two physicians is required.
- Parts of s-DS can be performed by a s-DS nurse with delegated tasks such as 1) Monitoring and documentation of vital parameters. 2) Administration of drugs according to physicians´ prescription.
- To fulfill the role in s-DS the nurse must have undergone and documented education and training in observation, airway management and pharmacology relevant to s-DS. If a s-DS nurse is locked into other tasks (e.g. sterile-dressed assistant at the table), there must be an additional s-DS-qualified physician or nurse present in the operating room.
- Anesthesiologic expertise must be available on immediate call to handle airway-related complications.

| **ASA classification** |  |
| --- | --- |
| No increased risk | 1 |
| Mild illness without functional limitation | 2 |
| Severe illness with functional limitation | 3 |
| Unstable/ decompensated life-threatening condition | 4 |

**Supplement S2: Simple Deep Sedation workflow**

1. Patient sitting on the operating table: Ensure uncomplicated airway status - SARI (Simplified Airway Risk Index) scores: Mouth opening, Mallampati scoring, Jaw- and Neck mobility.
2. With the patient supine on the operation table: Safe surgery time-out.
3. Continuous monitoring is established: ECG, blood oxygen saturation, respiratory curve (tidal movements), capnography.
4. Starting bolus Fentanyl
5. Airway: Facemask fitted with capnograph with flow 3-5l O _2_ /min from start
6. Noninvasive blood pressure every 2 minutes during induction until sedation is stable - then every 3 minutes.
7. Starting bolus Propofol. Slow continuous infusion through a peripheral venous catheter.
8. Infusion of propofol on the syringe pump starts directly following the initial bolus of propofol.
9. When the patient is asleep, supplemental propofol bolus is given as needed until oropharyngeal airway device is accepted.
10. During the sleep-initiation phase until the oropharyngeal airway is in place and respiration is in steady state, the patients´ groin can be prepared, sterile coverage applied, and puncture initiated.
11. An oropharyngeal or nasal airway is placed, free spontaneous ventilation without airway obstruction is ensured, and non-humidified oxygen is fitted with airway access.
12. Sedation is stable at RASS score -4 when the oropharyngeal airway is accepted without coughing. Only then is groin puncture (pain stimulus) performed.
13. The infusion rate during the procedure is guided by blood pressure, alveolar ventilation by capnography curve, arterial oxygen saturation and respiratory quality (rate, depth, stability)
14. Propofol infusion is stopped 5 minutes before expected end of procedure.
15. Waking up in the cath. lab. until awake with free breathing, and ability to actively assist in transferring from operating table to bed RASS score -1.
16. The patient is scored according to the RASS scale and the Danish Anesthesiologic society (DASAIM) guidelines for ability to discharge from intensive observation to normal ward/ standard observation. The responsible s-DS nurse accompanies the patient and conducts verbal handover to the nursing staff in the ward. Monitoring of the patient as usual after ablation for cardiac arrhythmias.

Respiration

- Pulse oximetry with addition/regulation of nasal O_2_ supplementation to keep **O_2_ saturation ≥ 93%**
- Quality of respiration (deep/shallow; even/uneven)
- Capnography of expiratory CO_2_ reflects alveolar ventilation with few seconds latency

Blood Pressure. Measured non-invasively with arm cuff every 3 minutes

- Mean arterial blood pressure **(MAP) ≥ 60 mm Hg** (main “driver” of vital organ perfusion)
- **Systolic BP ≥ 90 mm** Hg

Blood pressure decrease requiring treatment is regulated by:

1. Reduced propofol infusion rate and/or volume therapy (infusion of isotonic saline)
2. If this is not sufficient, pharmacological stimulation of blood pressure may be necessary:

- If vasoconstriction is required:

-Intravenous **phenylephrine/ metaoxedrine** 100 to 200 microgram boluses - eventually changed to continuous infusion via syringe pump if necessary or repeated dosing required.

- If chrono-inotropy is required:

-Intravenous **ephedrine** 5 mg to 10 mg boluses can be repeated every 3 minutes to max 30 mg.

**Supplement S3: Simple Deep Sedation drug dosing:**

|  | Starting dose | Bolus | Maintenance |
| --- | --- | --- | --- |
| Fentanyl | 25 - 50 µg | 25 - 50 µg | Occasionally necessary in minor doses of 25 – 50 µg. |
| Propofol | 0.6 mg/kg | 0.2-0.4 mg/kg | 5-10 mg/kg x hour (continuous infusion pump) |

**(ALWAYS 10 mg/ml)** bolus and maintenance via syringe pump.

| Weight (kg) | Start Bolus (mg) | Start Bolus  (ml) * | Basic maintenance  5 mg/kg/hour  (ml/hour) | Max. maintenance  10 mg/kg/hour  (ml/hour) | Supplement  Bolus (ml) |
| --- | --- | --- | --- | --- | --- |
| 50 | 30 | 3 | **25** | **50** | 2 |
| 55 | 33 | 3 | **28** | **55** | 2 |
| 60 | 36 | 4 | **30** | **60** | 2 |
| 65 | 39 | 4 | **33** | **65** | 2 |
| 70 | 42 | 4 | **35** | **70** | 2 |
| 75 | 45 | 5 | **38** | **75** | 2 |
| 80 | 48 | 5 | **40** | **80** | 2 |
| 85 | 51 | 5 | **43** | **85** | 3 |
| 90 | 54 | 5 | **45** | **90** | 3 |
| 95 | 57 | 6 | **48** | **95** | 3 |
| 100 | 60 | 6 | **50** | **100** | 3 |
| 105 | 63 | 6 | **53** | **105** | 3 |
| 110 | 66 | 7 | **55** | **110** | 3 |

*) In patients 70 years of age or older, the starting bolus is reduced by 1 ml.

No fixed reduction in maintenance or subsequent boluses but adapted to the patient's individual needs.

**Dosing of Phenylephrine/ Metaoxedrine to vasoconstrict/ increase MAP**

- Bolus – for temporary drop in blood pressure

[**Strong** solution: 1 mg/ml] give 0.1- 0.2 ml (100 µg – 200 µg)

- If repeat bolus injections are required despite primary measures of volume infusion and intracardiac pacing change to infusion:

[**Weak** solution: 0.1 mg/ml] Usually between 2 and 10 ml/hour via infusion pump (corresponding to 200 µg/hour to1000 µg/hour)

**Supplement S4. Ablation systems used in Simple Deep Sedation study phases 1 and 2**

| **Pulsed Field ablation “point-by point”** | Centauri ® Generator [Cardiofocus Inc Marlborough, MA USA]. Ablation energy delivered through irrigated Smarttouch ® catheters [Biosense Webster Inc, Irvine, Ca, USA] |
| --- | --- |
| **Pulsed field ablation “Single shot”** | Farastar ® Generator. Ablation energy delivery through Farawave ® Catheter (Boston Scientific, Inc Marlborough, MA, USA] |
| **Radio frequency ablation**  **“point-by-point”** | Smartablate Generator ® and ablation catheters Smarttouch SF ®, Thermocool ® and Celsius ® catheters [Biosense Webster Inc., Irvine, CA, USA] |
| **Radio frequency ablation**  **“Single shot**” | nGEN ® Generator. Ablation energy delivered by the Heliostar ® RFA balloon [Biosense Webster Inc., Irvine, CA, USA] |
| **Cryo ablation**  **“Single shot”** | CryoConsole ®. Ablation energy delivered by Arctic Front Advance ® 28 mm Balloon [ Medtronic Inc., Minneapolis, MN, USA] |
